# Supplementary material for: Differentiation of Human-Induced Pluripotent Stem Cell-Derived Endocrine Progenitors to Islet-like Cells Using a Dialysis Suspension Culture System
Source: Cells. 2021 Aug 7;10(8):2017. doi: 10.3390/cells10082017 (PMC8392085; doi:10.3390/cells10082017)
Supplement: Supplementary file 1 [file cells-10-02017-s001.zip › cells-1225222-supplementary.pdf]

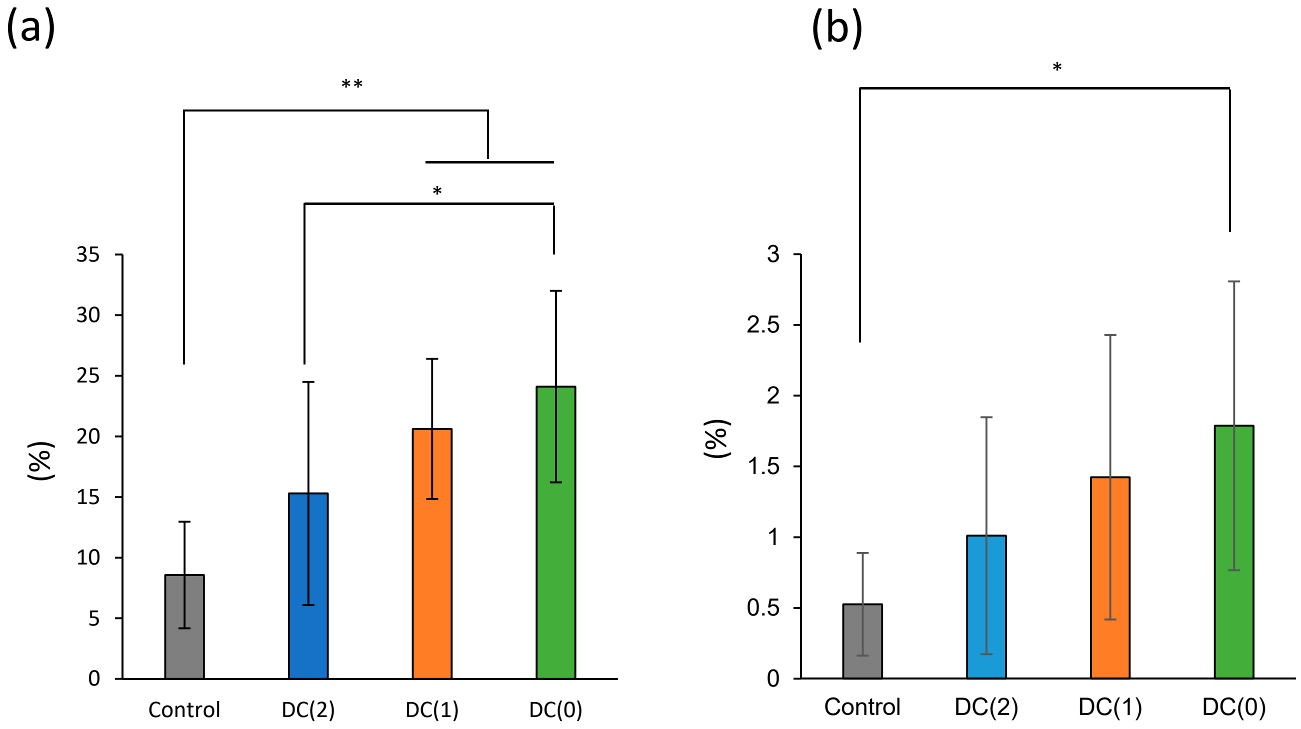

Figure supplement. 1 Percentage of positive cell area to islet-like cells cross-sectional area. (a) Rate of insulin positive area, (b) Rate of glucagon positive area. Control: without dialysis, medium change 2 times, DC(2) : with dialysis, medium change 2 times, DC(1) : with dialysis, medium change 1 time, DC(0) : with dialysis, without medium change. Data represent mean ± SD of (a) n = 12, (b) = 8 - 12 from two independent experiments. Statistical analysis as determined by one-way ANOVA with post hoc Tukey HSD test. (*$p < 0.05$,**$p < 0.01$)

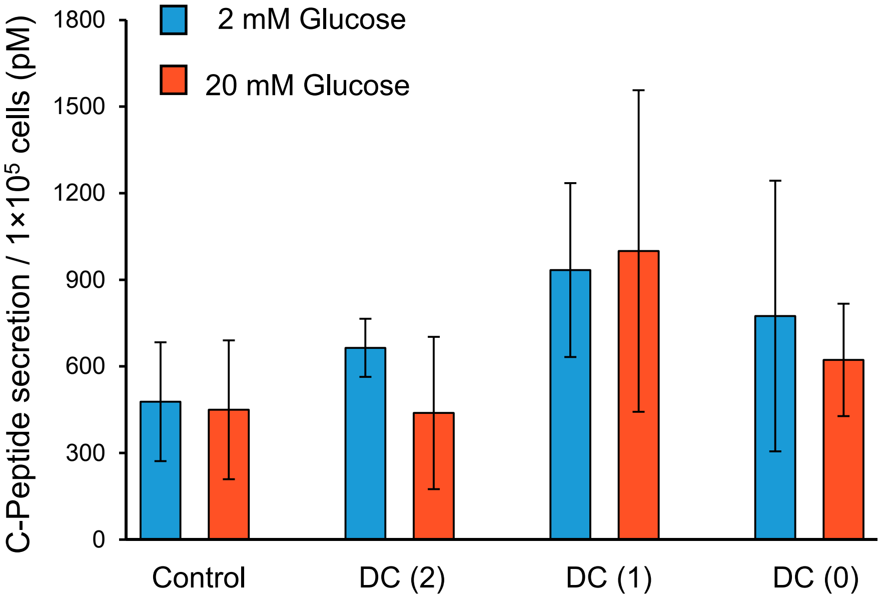

Figure supplement. 2 Glucose-stimulated C-peptide secretion of differentiated islet-like cells in first phase glucose stimulation. Data represent mean ± SD of n = 5 - 6 from two independent experiments.

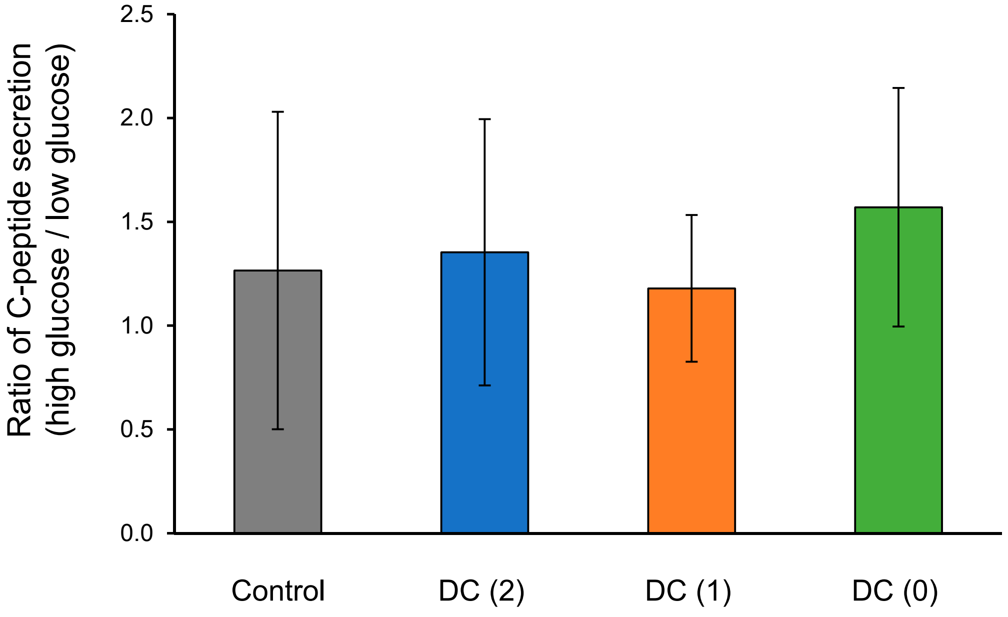

Figure supplement. 3 Fold-increase of C-peptide secretion response to the change of glucose concentration of second phase of glucose stimulation. Data represent mean ± SD of ± SD of n = 5 - 6 from two independent experiments.

Table supplement. 1 Primer sequences used in RT-qPCR

Gene	Primer sequence
NGN3	f_TAAGAGCGAGTTGGCACTGAG
	r_AGGGAGAAGCAGAAGGAACAAG
PDX1	f_CTTGGAAACCAACAACTATTCAC
	r_ATTAAGCATTTCCCACAAACA
NKX6.1	f_TCAACAGCTGCGTGATTTTC
	r_CCAAGAAGAAGCAGGACTCG
MAFA	f_CGCCGTATGAGTTCTGTG
	r_GGTGATCCTCTTCTGCTTC
INS	f_CTCCAGGACAGGCTGCATCA
	r_GGGCTGCGTCTAGTTGCAGT
GCG	f_AGGCAGACCCACTCAGTG
	r_CTAGAGTTTGACCAGCCACTCTC
SST	f_GGACAGATCTTCAGGTTCCAGG
	r_GGACAGATCTTCAGGTTCCAGG
GLUT1	f_TTGCAGGCTTCTCCAACTGGAC
	r_CAGAACCAGGAGCACAGTGAAG
GLUT2	f_TGGGCTGAGGAAGAGACTGT
	r_CCCATCAAGAGAGCTCCAACT
GCK	f_CACTGCTGAGATGCTCTTCGAC
	r_CCACGACATTGTTCCCTTCTG
PCSK1	f_TGATCCCACAAACGAGAACAAAC
	r_TGTGATTATTTGCTTGCATGGCA
B-actin (house keeping)	f_TTGCCAATGGTGATGACCTGG
	r_CCTCATGAAGATCCTCACCGA

Components	Cat. No.	Size	Price (USD)*	Stock		
				Concentration	Size	Price (USD/mL)
・Medium						
RPMI-1640	189-02025	500 mL	13.5		500 mL	0.027
DMEM-high glucose	043-30085	500 mL	13		500 mL	0.026
Advanced DMEM	12491-015	500 mL	34		500 mL	0.068
・Growth factors, Macromolecules						
Human recombinant FGF2	RCHEOT003	250 µg	858	50 µg/mL	4.75 mL	180.63
Human recombinant BMP4	130-098-787	1 mg	6,760	20 µg/mL	50 mL	135.2
Human recombinant Activin A	338-AC-050	50 µg	1,670	100 µg/mL	0.5 mL	3,340
Human recombinant FGF7	130-097-175	25 µg	600	50 µg/mL	0.5 mL	1,200
Human recombinant FGF10	069-06051	25 µg	424	50 µg/mL	0.5 mL	848
Human recombinant HGF	130-103-437	100 µg	1,740	50 µg/mL	2 mL	870
Human recombinant IGF	100-11	1 mg	250	50 µg/mL	20 mL	12.5
Bovine serum albumin (BSA)	68700-1KG	1 kg	4,000	50 mg/mL	20 L	0.2
Exendin4 (EX4)	050-07261	1 mg	400	50 µg/mL	20 mL	20
・Other chemicals						
Penicillin-streptomycin-amphotericin B suspension (PSA)	161-23181	100 mL	56	100×	100 mL	0.56
Non-essential amino acids (NEAA)	139-15651	100 mL	28	100×	100 mL	0.28
100 mmol/L Sodium pyruvate	190-14881	100 mL	18.5	100 mM	100 mL	0.185
CHIR99021	034-23103	5 mg	400	3 mM	3.582 mL	111.67
2-mercaptoethanol (2-ME)	21985023	50 mL	179	1000 ×	50 mL	3.58
Knockout serum replacement (KSR)	10828010	500 mL	566	100 ×	500 mL	1.132
B-27	17504044	100 mL	2,578	100 ×	100 mL	25.78
EC23	SC-361174	25 mg	297	5 mM	15 mL	19.8
Dorsomorphin	047-33763	5 mg	275	10 mM	1.0585 mL	259.80
SANT1	197-16351	5 mg	232	2.5 mM	5.355 mL	43.32
SB431542 (SB)	192-16541	25 mg	760	10 mM	6.5 mL	116.92
Indolactam V (ILV)	14647	1 mg	445	3 mM	1.1 mL	404.55
Alk5 inhibitor II (RepSox)	R0158-5MG	25 mg	1,080	5 mM	17.405 mL	62.05
200 mmol/L L-glutamine	073-05391	100 mL	31	200 mM	100 mL	0.31
Forskolin	063-02193	25 mg	300	10 mM	6.09 mL	49.26
Nicotinamide	N0636-100G	100 g	63	500 mM	1.64 L	0.04

* 100 JPY was calculated as 1 USD

Table supplement. 2 Price of components for differentiation medium. Price per volume of stock was calculated by the capacity of preparable stock volume.

	Stage 1		Stage 2		Stage 3		Stage 4		Stage 5		Stage 6		
	Required volume of stock for 2 L of medium (mL)	Cost (USD)	Required volume of stock for 2 L of medium (mL)	Cost (USD)	Required volume of stock for 2 L of medium (mL)	Cost (USD)	Required volume of stock for 2 L of medium (mL)	Cost (USD)	Required volume of stock for 2 L of medium (mL)	Cost (USD)	Required volume of stock for 2 L of medium (mL))	Cost (USD)	Total cost (USD)
・Medium													
RPMI 1640	6967.6	188.13	1898	51.25									239.37
DMEM high glucose					3854.664	100.22	3854.4	100.21					200.44
Advanced DMEM									3855.04	262.14	5701.8	387.72	649.87
・Growth factor, Macromolecules													
FGF2	2	361.26	2	361.26	4	722.53					1.2	216.76	1,661.81
BMP4	2	270.40									3	405.60	676.00
Activin A	6.4	21,376											21,376.00
FGF7			2	2,400									2,400.00
FGF10							4	3,392					3,392.00
HGF											6	5,220	5,220.00
IGF1											6	75	75.00
BSA	800	160.00	10	200.00									360.00
EX-4									4	80.00	6	120	200.00
													Sum: 35360.81
・Other chemicals													
PSA	32	17.92	8	4.48	16	8.96	16	8.96	16	8.96	24	13.44	62.72
NEAA	80	22.40	20	5.60	40	11.20	40	11.20					50.40
Sodium pyruvate	80	14.80	20	3.70									18.50
CHIR99021	2	223.34											223.34
2-ME	8	28.64											28.64
KSR	20	22.64											22.64
B-27			40	1031.20	80	2062.40	80	2062.40	80	2062.40	120	3093.60	10,312.00
EC23					0.536	10.61	0.4	7.92	0.16	3.17			21.70
Dorsomorphin					0.4	103.92	0.4	103.92	0.4	103.92			311.76
SANT1					0.4	17.33	0.4	17.33	0.4	17.33			51.99
SB					4	467.69							467.69
ILV							0.4	161.82					161.82
RepSOX							4	248.20	4	248.20	6	372.31	868.72
L-glutamine									40	12.40	60	18.60	31.00
Forskolin											6	295.57	295.57
Nicotinamide											60	2.30	2.30
Total	8000	22,685.53	2000	4,057.49	4000	3,504.86	4000	6,113.97	4000	2,798.53	6000	10,220.90	49,381.27

Table supplement. 3 Required cost for differentiation medium in each differentiation stage. Total cost to prepare 2 L of differentiation medium for whole stage was 49,381.27 USD and 71 % (35360.81 USD) of total cost was occupied by the cost of growth factors and macromolecules.
